# Health care provider decision-making and the quality of maternity care: An analysis of postpartum care in Kenyan hospitals

**DOI:** 10.1016/j.socscimed.2023.116071

**Published:** 2023-08

**Authors:** Dan Han, Emma Clarke-Deelder, Nora Miller, Kennedy Opondo, Thomas Burke, Monica Oguttu, Margaret McConnell, Jessica Cohen

**Affiliations:** a Lee Kuan Yew School of Public Policy, National University of Singapore, Singapore; b Department of Global Health and Population, Harvard T. H. Chan School of Public Health, Boston, MA, USA; c Department of Epidemiology and Public Health, Swiss TPH and University of Basel, Basel, Switzerland; d Kisumu Medical and Education Trust, Kisumu, Kenya; e Global Health Innovation Laboratory, Department of Emergency Medicine, Massachusetts General Hospital, Boston, MA, USA; f Harvard Medical School, Boston, MA, USA

**Keywords:** Provider decision-making, Postpartum care, Quality, Kenya

## Abstract

Evidence suggests that health care providers' non-adherence to clinical guidelines is widespread and contributes to poor patient outcomes across low- and middle-income countries. Through observations of maternity care in Kenya, we found limited adherence to guideline-recommended active monitoring of patients for signs of postpartum hemorrhage, the leading cause of maternal mortality, despite providers' having the necessary training and equipment. Using survey vignettes conducted with 144 maternity providers, we documented evidence consistent with subjective risk and perceived uncertainty driving providers' decisions to actively monitor patients. Motivated by these findings, we introduced a simple model of providers' decision-making about whether to monitor a patient, which may depend on their perceptions of risk, diagnostic uncertainty, and the value of new information. The model highlights key trade-offs between gathering diagnostic information through active monitoring versus waiting for signs and symptoms of hemorrhage to manifest. Our work provides a template for understanding provider decision-making and could inform interventions to encourage more proactive obstetric care.

## Highlights

• Postpartum hemorrhage (PPH) is the leading cause of maternal mortality.
• Providers in three Kenyan hospitals performed little monitoring for signs of PPH.
• Survey vignettes uncover links between subjective risk, uncertainty and monitoring.
• A simple model draws from these links to explain under-monitoring.
• The model also illuminates key tradeoffs between monitoring and wait-and-see.

## 1 Introduction

Across low- and middle-income countries (LMICs), suboptimal health care quality is estimated to cause millions of excess deaths each year (Chou et al., 2019; Kruk et al., 2018a). Diagnostic errors – missed, inappropriately delayed, or incorrect diagnoses – are considered particularly harmful (Singh et al., 2017; WHO, 2016). Evidence-based guidelines generally call for assessment of all relevant information during the diagnostic process. However, non-adherence to guidelines related to asking diagnostic questions and ordering tests in cases with indicated symptoms is ubiquitous (Das and Hammer, 2014; Krüger et al., 2017; Kruk et al., 2018b). Most approaches to improving provider guideline adherence in LMICs are premised on the assumption that non-adherence stems from a lack of facility infrastructure, inadequate provider knowledge, excessive workload, or low incentives (Das et al., 2018; Rowe et al., 2018). Yet, studies of the relationships between structural inputs, provider training, staffing, and quality of care have yielded mixed results (Kovacs and Lagarde, 2022; Leslie et al., 2017; Rowe et al., 2021; Souza et al., 2013), and growing evidence reveals large gaps between providers’ knowledge and actual practice of guideline-recommended care (Gage et al., 2018; Mohanan et al., 2015; Rokicki et al., 2021). Financial incentives, which are being deployed across LMICs to improve provider practice, have shown limited positive effects (Diaconu et al., 2021; Kovacs et al., 2020a). These findings suggest that a better understanding of the behavior and decision-making of health care providers may be a critical element toward improving quality of care in LMIC contexts. Even in settings with clear guidelines, providers exert discretion over clinical decisions, during which their beliefs, perceptions, and intuition likely play important roles.

Non-financial behavioral factors driving suboptimal clinical decisions in LMICs remain poorly understood. Through vignettes and analysis of detailed clinical records, studies in high-income settings suggest that providers may deviate from guidelines due to factors such as aversion to missed diagnoses and responsiveness to salient symptoms (Blumenthal-Barby and Krieger, 2015; Chan et al., 2022; Cutler et al., 2019; Meyer et al., 2013; Mullainathan and Obermeyer, 2022). When providers in LMICs are asked to identify the reasons why guidelines are not followed, many point to conventional explanations such as knowledge, resources, and staffing (Braddick et al., 2016; Lange et al., 2014; Seyoum et al., 2021). This may be consistent with providers’ lack of self-awareness of how their decision-making affects quality of care (Cassam, 2017). Experimental approaches to understanding provider behavior and decision-making may complement approaches that ask providers to self-report the reasons for poor performance. Furthermore, most prior evidence explores non-adherence to guidelines in routine care settings. More evidence is needed from emergency settings, where provider decision-making may be particularly consequential but also more susceptible to misjudgments due to the fast pace and higher degree of uncertainty.

In this study, we explored health care providers' decision to actively monitor for early signs and symptoms of postpartum hemorrhage (PPH). PPH, an obstetric emergency characterized by excessive bleeding after childbirth, is the leading preventable cause of maternal deaths in LIMCs (Say et al., 2014). To ensure timely diagnosis of PPH, guidelines emphasize regular, active monitoring for various signs, symptoms, and potential causes of excessive bleeding for all postpartum women during the first 24 h after childbirth (WHO et al., 2015). In Kenya, the setting of our study and a country with 342 maternal deaths per 100,000 live births in 2017 (WHO et al., 2019), PPH causes an estimated one-fifth of maternal deaths (Ministry of Health Kenya, 2017). Delays in starting treatment and inadequate monitoring are found to be two of the top three provider-related factors contributing to maternal deaths from hemorrhage (Ministry of Health Kenya, 2017).

Our study focused on how providers' risk perceptions, diagnostic uncertainty, and the value of information influence provider decision-making. PPH is a novel and important domain for studying subjective judgement under uncertainty and information-seeking. First, diagnosing PPH is a complex process due to the possibility of hidden bleeding, a lack of accurate methods to quantify blood loss, and the heightened susceptibility to blood loss in women with certain underlying conditions such as anemia (Bienstock et al., 2021; Borovac-Pinheiro et al., 2018; Diaz et al., 2018; Pacagnella et al., 2013). This means that a timely diagnosis often requires the integration of multiple sources of information, none of which may be highly specific or predictive on its own. Second, although studies have identified important pregnancy- and labor/delivery-related factors to facilitate early risk assessment, many women develop PPH in the absence of recognized risk factors, suggesting the dynamic nature of PPH risk and the need for continuous assessment of a patient's condition (Ende et al., 2021; Erickson et al., 2020).

We began by presenting evidence of limited and inconsistent provider adherence to postpartum monitoring guidelines in the maternity departments of three large Kenyan hospitals, drawing on data collected during observations of over 500 vaginal deliveries. We then reported the results of survey vignettes conducted with 144 maternity care providers from the study facilities to explore how subjective risk and perceived uncertainty influence the decision to monitor. The vignettes showed that providers' assessment of PPH risk varied substantially across providers and that the relationship between subjective PPH risk assessment and known risk factors was weaker than expected. Most providers chose not to actively monitor when their subjective PPH risk was low, regardless of the risk factors present. Importantly, we found that providers' perceived uncertainty, captured by their specifying a range of possible values when assessing PPH risk for a given vignette as opposed to submitting a single risk value, was an important driver of active monitoring. However, the link between uncertainty and monitoring was nonexistent among subjectively low-risk cases, consistent with monitoring not being useful when providers believed PPH could be readily ruled out. Together, these findings point to two possible explanations for under-monitoring. First, providers may underestimate PPH risk, thus ruling out PPH for most patients soon after delivery. Second, providers may not monitor because they do not perceive uncertainty in risk assessment.

Building on these findings, we introduced a model to describe the provider's decision-making process and highlight key trade-offs that may affect the decision to perform active monitoring. The model is premised on the idea that diagnostic uncertainty – a situation where a provider cannot ascertain a patient's PPH risk – prevents her from confirming or ruling out PPH without new information. In the model, the provider determines whether she needs additional information to resolve uncertainty in her assessment of a patient's PPH risk; if she needs information, she decides whether to collect information through active monitoring or to wait for information that may be revealed when clinical signs present themselves (“wait-and-see”), weighing the costs and benefits of these two alternatives based on her beliefs.

Our study joins the emerging literature on drivers of low quality of care in LMICs through a behavioral perspective (Engl et al., 2019; Kovacs et al., 2020b). Our findings highlight that policy instruments to improve adherence to postpartum monitoring guidelines should incorporate a broader lens and consider why providers do not perceive uncertainty or information needs in the diagnostic process (e.g., due to overconfidence), how they respond to diagnostic uncertainty when it is present (e.g., active monitoring vs. wait-and-see), and how their beliefs (e.g., about how effective timelier treatment is and how informative monitoring could be) shape their decisions.

## 2 Setting and data

We observed clinical actions performed for vaginal deliveries in the maternity departments of three large hospitals in Nairobi and Western Kenya from October 2018 through February 2019 and conducted survey interviews with maternity care providers in these facilities from November 2019 through February 2020. Prior to data collection, the study team conducted semi-structured formative interviews with small purposive samples of maternity care providers from the study facilities to learn about PPH management practices in the local context.

The study facilities are regional public referral and teaching hospitals with roughly 4,500–14,000 vaginal deliveries each in 2018. In this context, most routine care is provided by nurse-midwives, with medical students playing a significant role in assisting with clinical duties. Medical doctors are primarily deployed in the case of emergencies, such as the need for a C-section or if a complication develops. Staffing is more limited during night shifts: on average, there are 2–3 patients per provider from 8 a.m. to 7 p.m. and 5–8 patients per provider from 8 p.m. to 7 a.m. the next morning. There are specific providers assigned to the postpartum ward, who are not responsible for patients in the admission area or labor ward.

Women who have just given birth usually stay in the labor and delivery ward for another hour as per the Kenyan guidelines. They are then transferred to the postpartum ward, where the main provider task is to monitor the condition of mothers and newborns and discharge patients to return home.

### 2.1 Direct observation of vaginal deliveries

Direct observations of deliveries were conducted in the study facilities by a team of Kenyan clinicians. The data collection tool was designed based on the World Health Organization and Kenyan clinical guidelines for labor, delivery, and postpartum care. Pregnant women who were aged 15 or over, delivered vaginally, and provided informed consent were included in the direct observations. Patients referred to the study facility from a lower-level facility before giving birth were also included. 907 vaginal deliveries met the inclusion criteria. The resulting data contains 1) patient characteristics recorded from the initial exam at admission and antenatal book and 2) clinical actions performed by maternity care providers at admission, during labor and delivery, and during the postpartum period and their timestamps. Clarke-Deelder et al. (2023) documents the full details of the delivery observations.

Our study sample was restricted to patients for whom the postpartum period was observed. The postpartum period is defined as the first 24 h after delivery. If a patient stayed in the facility for less than 24 h after delivery, her discharge from the facility marked the end of the postpartum period in the data. We excluded patients suspected of developing PPH within 15 min of delivery (Appendix Table A1). We imposed this sample restriction because monitoring in these cases would mainly serve the purpose of providing follow-up care after PPH treatment, whereas our interest lied in active monitoring as a means to detect PPH early. A patient was suspected to have PPH if a provider indicated concern with potentially excessive bleeding or took action to manage abnormal bleeding, as observed by the enumerators. About 9% of patients in our sample had a suspected PPH.

Analysis involving data on PPH risk factors was further restricted to the subset of patients with complete observation for the initial exam, labor and delivery, and the postpartum period. We selected and measured PPH risk factors based on the literature, inputs from clinicians on the research team, the Kenya national guidelines, and data availability (Appendix Table A2).

Table 1 provides an overview of the delivery observation samples. On average, patients stayed in the study facilities for 29 h after childbirth, but a small proportion (5–6%) left the facilities in less than 12 h after delivery. Roughly 45% of patients had at least one documented PPH risk factor (e.g., prolonged labor) and 9% had at least two.

**Table 1:** Summary statistics for the delivery observation sample.

Variables	Postpartum period observed		Admission through discharge observed	
	n (%) or mean (sd)	N	n (%) or mean (sd)	N
Maternal age (years)	25.5 (5.2)	589	25.4 (5.2)	515
Nulliparous	221 (36.3%)	608	193 (36.8%)	524
Referred from lower-level facilities	26 (4.3%)	608	23 (4.4%)	524
Companion present during the postpartum period	66 (12.7%)	520	59 (13.0%)	453
Time from delivery to discharge/end of observation (hours)	29.6 (20.6)	594	29.2 (20.2)	511
Discharged within 24 h after delivery	257 (43.3%)	594	229 (44.8%)	511
Discharged within 12 h after delivery	30 (5.1%)	594	30 (5.9%)	511
≥1 risk factor documented up to delivery*	272 (44.7%)	608	237 (45.2%)	524
≥2 risk factors documented up to delivery*	56 (9.2%)	608	50 (9.5%)	524
Individual risk factor:				
Multiple gestation	7 (1.2%)	591	5 (1.0%)	509
Antepartum hemorrhage	15 (2.6%)	588	15 (2.9%)	516
Hemoglobin <10 g/dL	76 (14.8%)	515	63 (14.0%)	450
Documented history of PPH	4 (1.2%)	341	3 (0.9%)	323
Documented history of C-section	8 (2.3%)	341	6 (1.9%)	322
Parity ≥5	12 (2.0%)	608	12 (2.3%)	524
Oxytocin exposure ≥10 IU	66 (11.1%)	595	53 (10.3%)	515
Received antibiotics (suspected infection)	7 (1.2%)	603	5 (1.0%)	519
Suspected prolonged labor	101 (19.9%)	507	97 (21.5%)	451
Birthweight ≥4 kg	37 (7.0%)	532	32 (6.9%)	463
Suspected retained placenta	4 (0.7%)	534	4 (0.9%)	457

Notes: Samples exclude suspected PPH cases identified within 15 min of delivery. The Ns for history of PPH and C-section only include women who had given birth before. *Missing risk factors are assumed to reflect absence of risk factors when calculating the total number of risk factors and, therefore, the number of risk factors may be underestimated.

### 2.2 Provider survey and vignettes

Surveys with maternity care providers were conducted by a team of trained Kenyan enumerators. During these interviews we assessed provider knowledge regarding postpartum monitoring guidelines. We also conducted vignettes to examine providers' subjective risk and perceived uncertainty in risk assessment and explored how they were correlated with the decision to actively monitor patients. The survey was administered to a convenience sample of 144 maternity care providers, including 100 qualified health workers and 44 medical students who had completed at least three weeks of maternity ward rotation at the time of the interview. Table 2 reports the summary statistics of the provider sample.

**Table 2:** Summary statistics for the provider interview sample.

	Qualified health workers		Students	
	n (%) or mean (sd)	N	n (%) or mean (sd)	N
Female	69 (79.3%)	87	26 (59.1%)	44
Age <25	14 (16.1%)	87	35 (79.5%)	44
Age 25–34	51 (58.6%)	87	7 (15.9%)	44
Age 35–44	12 (13.8%)	87	2 (4.5%)	44
Age ≥45	10 (11.5%)	87	0 (0.0%)	44
Working full time	71 (82.6%)	86	–	
Highest training: bachelor's degree or higher	26 (29.5%)	88	–	
Years of experience since highest training >5 years	29 (33.7%)	86	–	
Risk attitude (willingness to take risks in general)	7.1 (2.4)	100	6.8 (2.6)	44

Notes: We interviewed 100 qualified health workers and 44 medical students from the study facilities. The N for qualified health workers reported here is below 100 except for risk attitudes due to missing values. Risk attitude measures willingness to take risks in general using a 0–10 scale, with 0 = “completely unwilling” and 10 = “very willing.”

The knowledge questions were designed based on clinical guidelines. Providers were asked to indicate the monitoring frequencies after a normal, uncomplicated delivery and to list the health checks and patient care they would complete during postpartum monitoring. We recorded providers' unprompted responses to these questions.

The vignettes were designed based on actual patient profiles from the delivery observations. Providers were presented with multiple vignettes at once. Upon reviewing the cases, they were asked to assign each patient described in the vignette to one of seven beds on a stylized facility map designed to mimic the physical layout of the study facilities. They were also asked to specify the patient's risk of excessive bleeding on a scale of 0–10. To elicit perceived uncertainty about risk, providers were given the option to respond with either a single risk value (indicating no uncertainty) or a risk interval (a range of values with upper and lower bounds, indicating uncertainty). Our measure of uncertainty circumvents certain limitations of existing methods, such as directly asking how sure providers feel that their answer is correct (Moore and Schatz, 2017). Our approach is similar to the one used in Bachmann et al. (2020) to examine belief ambiguity. Considering that providers may default to a single risk value, we emphasized in our question that they could respond using either a range or a single number. The vignettes were pre-tested with five clinicians including two Kenyan clinicians. More details about the vignettes are provided in Section 5 and the Appendix.

## 3 Poor adherence to monitoring guidelines

**Fig. 1:**
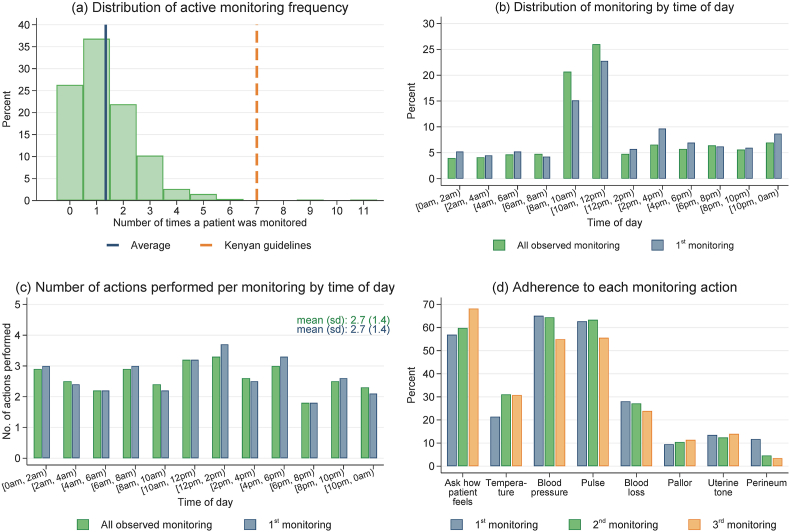
Patterns of postpartum monitoring based on direct observations Notes: Sample includes patients whose postpartum period was observed and excludes suspected PPH cases identified within 15 min of delivery. (a) shows the distribution of how many times a patient was monitored and its mean (solid line), along with guideline-recommenced monitoring frequency (dash line). (b) shows the distribution of monitoring by time of day for all observed monitoring and for the first monitoring a patient received. (c) shows the average number of guideline-recommended actions (out of eight) performed each time a patient was monitored for all observed monitoring and for the first monitoring received. (d) shows the percentage of monitoring during which each recommended action was performed. Checking pallor was recorded as completed if the provider used an appropriate method.

Our direct observations showed that active postpartum monitoring in the study facilities was exceptionally limited compared to Kenya's national guidelines. The Kenya guidelines recommend active monitoring of women every 15 min in the first hour after childbirth as well as at 1 h, 6 h, and before discharge (Ministry of Public Health and Sanitation & Ministry of Medical Services Kenya, 2012). In our data, one-quarter of patients were never monitored and less than 40% of patients were monitored more than once (Fig. 1a). Monitoring was primarily done between 8 a.m. and 12 p.m. during morning rounds but remained infrequent throughout the rest of the day (Fig. 1b). On average, providers performed three health checks out of eight guideline-recommended actions during each monitoring, and adherence to these actions did not vary considerably by time of day (Fig. 1c). The most consistently performed actions included asking how the patient was feeling, taking blood pressure, and assessing heart rate (Fig. 1d).

**Fig. 2:**
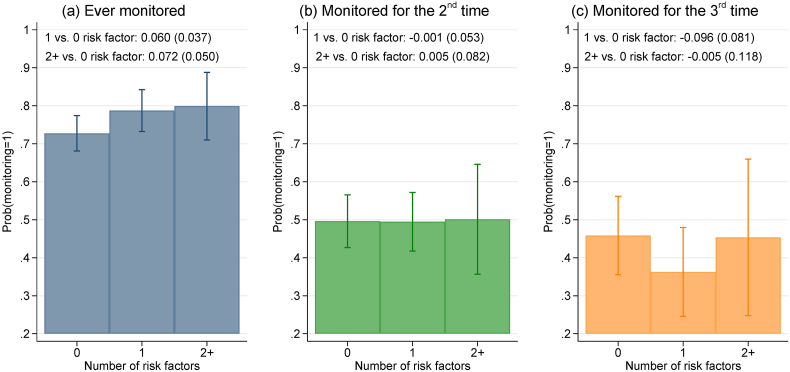
Probability of monitoring by risk factors documented up to delivery of the baby Notes: This figure reports the predictive margins and 95% confidence intervals based on linear probability models with robust standard errors using direct observation data. Regression-adjusted differences in monitoring probabilities relative to patients with 0 risk factor are reported at the top of each graph; standard errors in parentheses. The dependent variable is an indicator variable for whether a patient was ever monitored in (a), monitored for the 2nd time conditional on being ever monitored in (b), or monitored for the 3rd time conditional on being monitored twice in (c). The respective sample size for the three regressions is 520, 393, and 195. Regressions control for facility indicators, time of delivery (0–5am, 6–11am, 12-5pm, 5–11pm), weekend births, and an indicator for referral cases. Sample includes deliveries with complete observation from admission through discharge and excludes suspected PPH cases identified within 15 min of delivery.

Given potential staffing constraints and limited attention, providers may target monitoring to patients with multiple recognized risk factors. Indeed, patients with two or more risk factors experienced suspected PPH at a rate more than twice that among patients without documented risk factors (Appendix Table A3). However, we saw limited evidence that monitoring was inconsistently performed across patients because monitoring resources were targeted to those at higher risk. Fig. 2 shows that the probabilities of ever being monitored and being monitored more than once were not statistically significantly higher among patients with documented PPH risk factors.

Consistent with previous literature, we found that structural factors and provider knowledge alone could not explain the low monitoring rates. Facility audits suggested that all three maternity departments had necessary monitoring equipment such as blood pressure cuffs and thermometers as well as treatment for PPH in stock (Clarke-Deelder et al., 2023). When surveyed, providers in these facilities demonstrated knowledge of postpartum monitoring consistent with the Kenyan and international guidelines in terms of monitoring timing, frequency, and the health checks to perform during monitoring (Table 3). On average, providers indicated that a woman should be monitored every 22 min in the 1st hour after delivery and every 1.6 h thereafter following a normal, uncomplicated birth. Regarding guideline-recommended monitoring actions, over 90% of providers mentioned checking vital signs and estimating blood loss, and two-thirds and close to 60% of providers mentioned assessing for perineal tears and checking uterine contractions, respectively.

**Table 3:** Provider knowledge about postpartum monitoring.

	All providers	Qualified health workers	Students
	mean (sd) or n (%)		
***During the 1st hour following a normal, uncomplicated delivery***			
Monitoring frequency (every __ minutes)	21.5 (8.3)	22 (8.7)	20.6 (7.4)
Should monitor at least once every 15 min	78 (54.2%)	53 (53.0%)	25 (56.8%)
Should monitor at least once every 30 min	143 (99.3%)	99 (99.0%)	44 (100%)
***After the 1***st ***hour following a normal, uncomplicated delivery***			
Monitoring frequency (every __ hours)	1.6 (1.4)	1.6 (1.5)	1.4 (1.3)
Should monitor at least once every 1 h	91 (63.2%)	59 (59.0%)	32 (72.7%)
Should monitor at least once every 2 h	115 (79.9%)	79 (79.0%)	36 (81.8%)
***Monitoring actions in the 1***st ***hour after delivery***			
Estimate blood loss	135 (93.8%)	97 (97.0%)	38 (86.4%)
Take vital signs	133 (92.4%)	93 (93.0%)	40 (90.9%)
Assess for tears and lacerations in the perineum	96 (66.7%)	70 (70.0%)	26 (59.1%)
Check uterine contraction	85 (59%)	68 (68.0%)	17 (38.6%)
Check pallor	23 (16%)	19 (19.0%)	4 (9.1%)
No. correct response out of 5	3.3 (1)	3.5 (0.9)	2.8 (1)
Correctly identify 4 or more monitoring actions	66 (45.8%)	54 (54.0%)	12 (27.3%)
N	144	100	44

Notes: Data based on surveys with providers from the study facilities. The monitoring frequency question asked providers how often a women should be monitored after a normal, uncomplicated delivery. The knowledge question asked providers to list the monitoring actions they would perform for this woman within 1 h after delivery. Spontaneous responses to these questions were recorded.

The low monitoring rates in these relatively well-equipped facilities with trained providers presented a puzzle that motivated a closer examination of providers' decision-making processes. Specifically, we focus on the role of two factors in shaping the provider's decision to actively monitor a patient: 1) subjective risk i.e., the provider's perceived probability that a patient has PPH, and 2) diagnostic uncertainty, the presence of which implies information gaps and recognition of the value of information in impacting subsequent decisions. In the next section, we explore these relationships using results from survey vignettes.

## 4 Relationship between risk, uncertainty and monitoring

### 4.1 Vignette setup

We developed five vignettes describing hypothetical patients based on patient characteristics in the delivery observations and relevant literature on PPH risk factors. Each provider was given four vignettes, including Vignettes 1 to 3 plus one vignette chosen randomly from the remaining two (Vignette 4 or 5). We initially assigned the vignettes this way to explore if changing the patient mix may affect active monitoring decisions. For this analysis, we pooled together all data instead of stratifying them by whether a provider was assigned Vignette 4 or Vignette 5. The PPH risk factors presented in each vignette were as follows (more details in Appendix).

Vignette 1 (Low Risk): Normal labor & delivery.

Vignette 2 (High Risk): Mild anemia, a history of PPH, birthweight >4 kg.

Vignette 3 (Elevated Risk): Mild anemia, antepartum hemorrhage, oxytocin exposure during labor ≥10 IU.

Vignette 4 (High Risk): Oxytocin exposure during labor ≥10 IU, prolonged labor.

Vignette 5 (Elevated Risk): Mild-to-moderate anemia, birthweight >4 kg.

By including risk factors that differ in terms of how “salient” they might appear from a provider's perspective, these vignettes were designed to represent different levels of PPH risk. Vignette 1 was a relatively low-risk case due to a lack of risk factors; Vignettes 2 and 4 had relatively high risk due to the presence of a history of PPH and prolonged labor, respectively – both are significant risk factors (Ende et al., 2021). Vignettes 3 and 5 represented cases with elevated PPH risk: anemia among pregnant women and oxytocin use to augment or induce labor are relatively common in the study setting (Table 1), and there is greater uncertainty regarding the link between the other presented risk factors (antepartum hemorrhage, high birthweight) and PPH (Ende et al., 2021).

PPH risk was captured using a 0–10 risk scale, with the option to indicate uncertainty using a risk interval (e.g., 6–8 instead of 6). Intention to monitor was captured based on patient bed assignment using a facility map (see Appendix). The indicator for active monitoring equals 1 if providers assigned the patient to one of the two high-priority beds in the post-delivery care unit (i.e., Beds 1 and 2 on the map) and 0 otherwise. Providers could assign one patient to each bed: this was to reflect the capacity constraints in our setting and address the concern that providers may otherwise overstate their intention to monitor because of experimenter demand effects.

### 4.2 Vignette analysis and results

**Fig. 3:**
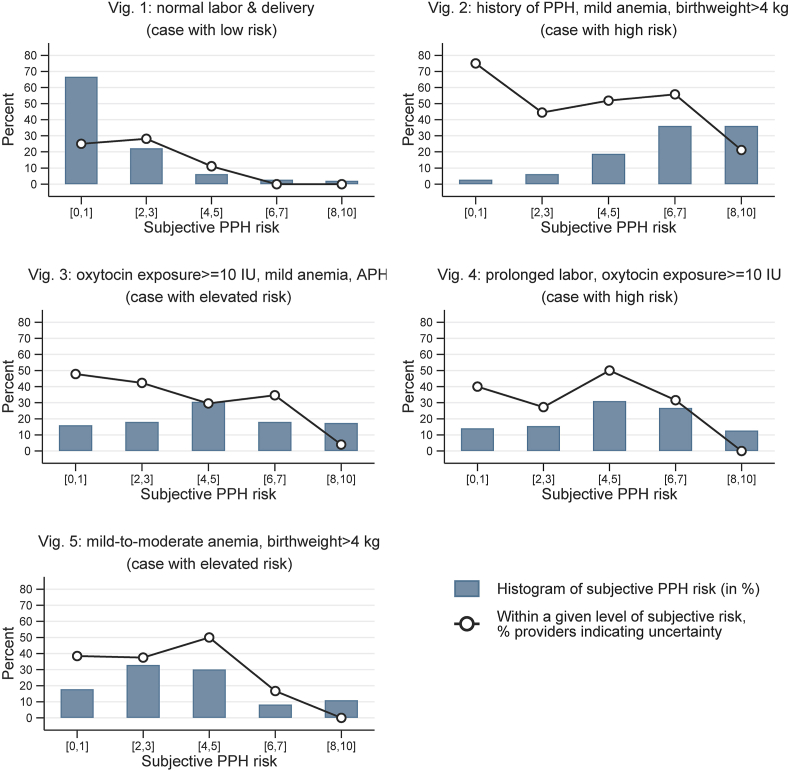
Subjective PPH risk (bar) and indicated uncertainty (circle) in vignettes Notes: Data from survey vignettes. APH = antepartum hemorrhage. Subjective PPH risk was measured using a 0–10 scale. If a provider specified a risk interval, we assigned their responses to one of the five subjective risk brackets based on her risk interval's lower bound.

Fig. 3 shows the distribution of providers' subjective PPH risk and the percentage of providers indicating uncertainty at a given level of PPH risk for each vignette. We coarsened providers’ subjective PPH risk into five brackets: [0, 1], [2, 3], [4, 5], [6, 7], [8, 10]. For responses specifying risk intervals instead of single values, we determined the bracket based on the lower bound of the interval (for example, if a provider specified a risk interval of 5–7, her response would be assigned to bracket [4, 5]).

As Fig. 3 indicates, most providers agreed with each other on their assessment for Vignette 1 (normal labor & delivery) and Vignette 2 (a history of PPH), which were the more “obvious” cases by design. However, their judgements varied considerably for Vignettes 3–5. For example, one-third of providers considered the risk of PPH for Vignette 3 to be between 0 and 3, while another third considered it to be between 6 and 10. In addition, over half of the providers who considered Vignettes 3–5 as low risk cases felt certain about their own judgement. Such variability in PPH risk assessment is notable given that, as mentioned earlier, only 9% of patients in our delivery observations had two or more PPH risk factors and these patients also experienced a much higher PPH rate.

Overall, just above half of the providers indicated uncertainty in risk assessment for any vignette. Regression analysis of the associations between provider characteristics and their propensity to indicate uncertainty shows that older providers were more likely to indicate uncertainty (Appendix Figure A1). However, because age and experience are correlated, the estimated coefficient on age may have captured the association between experience (and other correlated but unobserved characteristics) and uncertainty. On the other hand, scoring higher on questions about monitoring guidelines was not associated with indicated uncertainty.

Next, we pooled together all vignettes to explore the associations between risk, uncertainty, and monitoring using a linear probability model. We regressed the provider's monitoring decision on her subjective PPH risk, a binary variable for uncertainty (equaled 1 if the provider reported a risk interval and 0 if they reported a single risk), the interaction term of these two variables, and vignette and provider fixed effects. We performed two robustness checks. In the first check, we coarsened providers' subjective risk based on the midpoint of the risk interval. In the second, we relaxed the constraint on the number of beds indicating intention of monitoring and recoded the monitoring variable such that it equaled 1 if a patient was assigned to a bed in the post-delivery care unit (Bed 1 and 2) or the bed closest to the nurse station in the postpartum unit (Bed 7).

**Fig. 4:**
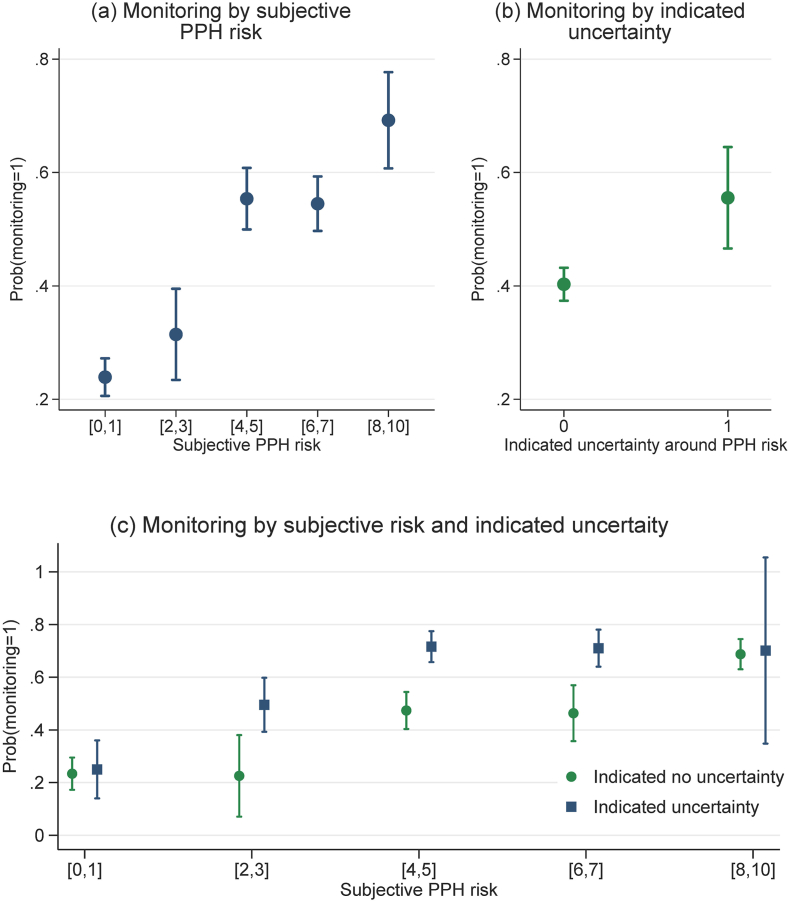
Predicted probabilities of monitoring by subjective risk and indicated uncertainty based on survey vignettes Notes: The figure above reports predictive margins based on this linear probability model: $y_{ij}=\alpha_{i}+\varphi_{j}+\sum \beta_{k}\times Risk\_Bracket_{ij}^{k}+\lambda \times Uncer{tainty}_{ij}+\sum \tau_{k}\times Ris{k\_Bracket}_{ij}^{k}*Uncer{tainty}_{ij}+v_{ij}$. Data from survey vignettes. The dependent variable is an indicator for whether provider i chose to monitor the patient presented in vignette j. Subjective PPH risk was measured using a 0–10 scale; If a provider reported a risk interval, we assigned their responses to one of the five risk brackets based on her risk interval's lower bound. All regressions are adjusted for vignette fixed effects $\varphi_{j}$ and provider fixed effects $\alpha_{i}$. 95% confidence intervals are based on standard error clustered at the facility level. Wild cluster bootstrap yields similar results.

Fig. 4 presents the predicted probabilities of monitoring based on the regression analysis and Appendix Table A4 reports the adjusted differences in monitoring probabilities. On average, intention to monitor increased in subjective PPH risk (Fig. 4a). The probability of monitoring was 0.24 for vignettes in the lowest risk bracket and more than doubled for vignettes in the higher risk brackets (Appendix Table A4). Indicated uncertainty in PPH risk assessment was also correlated with monitoring (Fig. 4b). When providers felt certain about their assessment, the probability of monitoring for any given vignette was 0.4; perception of uncertainty was associated with a higher probability of monitoring by 15 percentage points (Appendix Table A4). Moreover, the relationship between indicated uncertainty and monitoring varied across subjective risk (Fig. 4c). For vignettes in the lowest and highest risk brackets, indicated uncertainty was not associated with monitoring. For vignettes in the three risk brackets in the middle, uncertainty was associated with a greater probability of monitoring by at least 20 percentage points (Appendix Table A4). The robustness checks yield similar results (Appendix Table A4).

To summarize, we found that, first, providers' risk perception was not as responsive to recognized risk factors as we had expected. Second, monitoring was correlated with subjective PPH risk, rather than objective risk factors. Third, monitoring was associated with perceived uncertainty about risk, implying information needs due to uncertainty. But this relationship was not observed for cases of low or high subjective risk. This suggests that PPH may be readily ruled out or confirmed in these cases, rendering information less valuable despite the presence of uncertainty.

In the next section, we introduce a simple model of provider decision-making consistent with these findings. Apart from the implication that a lack of uncertainty drives under-monitoring, which is directly connected to the vignettes, the model yields additional implications about the trade-offs between active monitoring versus “wait-and-see” conditional on uncertainty and information needs.

## 5 A model of active monitoring

**Fig. 5:**
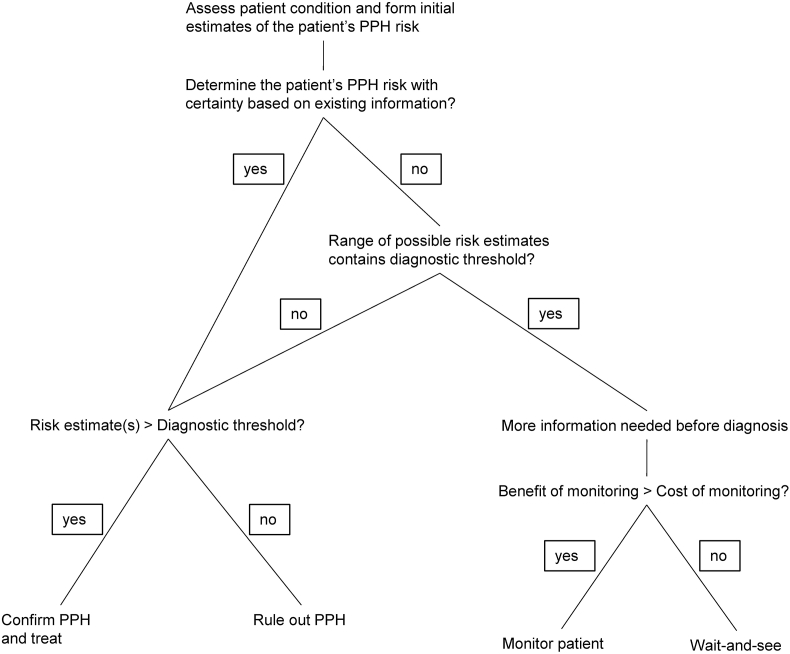
Decision-making process about whether to perform active monitoring.

We modeled the provider's decision-making process as follows (Fig. 5):

1. Following delivery, the provider estimates the probability that a patient has PPH.
2. If the provider feels certain about her assessment (or if she is uncertain but the range of possible risk estimates does not contain the diagnosis threshold), she makes a diagnosis confirming or ruling out PPH. Otherwise, she decides that additional information is necessary before a diagnosis.
3. Conditional on needing more information, the provider decides whether to monitor the patient by comparing the costs and benefits of actively seeking information against passively waiting for information to emerge.

We define uncertainty as a situation where people cannot ascertain the level of risk due to information gaps (Ellsberg, 1961). Uncertainty about PPH risk may stem from missing information (e.g., internal bleeding that is hard to observe) or ambiguous information (e.g., weak correlations between common maternal risk factors and PPH). When uncertainty is present, providers may prefer treatment – such as the use of therapeutic uterotonics and uterine massage – over doing nothing, as treatment helps to insure against the worst patient outcomes (Berger et al., 2013). However, treatment is costly given staffing and resource constraints. Active monitoring for signs and symptoms of PPH generates information that could reduce diagnostic uncertainty and may be less labor- and resource-intensive than treatment.

Alternatively, the provider can choose the “wait-and-see” approach, i.e., by waiting for strong signals of PPH including visible blood loss and patient deterioration. Allowing time to pass before taking action leads to more informative signals but can cause major delays in treatment and lower treatment success. “Wait-and-see” differs from active monitoring in that the provider makes no proactive effort to resolve uncertainty. Importantly, wait-and-see is empirically indistinguishable from situations where the provider has already ruled out PPH, so a lack of monitoring does not always imply an absence of uncertainty.

### 5.1 Model set up

There are two objective states θ∈{0,1}, where θ=1 indicates the patient has PPH and θ= 0 indicates she does not have PPH. A patient's PPH state is determined by whether any underlying causes of PPH such as uterine atony or a high-degree laceration are present. Upon observing patient i, the provider forms an initial judgement of the patient's probability of PPH $p_{i}$. She then decides between treatment and no treatment based on whether $p_{i}$ exceeds a diagnostic threshold q.

When the provider cannot ascertain the patient's PPH risk, her initial assessment includes a range of possible ${p_{i}}^{\prime }s$, with $p_{i}^{L}$ and $p_{i}^{U}$ being the lower and upper bounds of the range. The need for new information arises when $p_{i}^{L}<q<p_{i}^{U}$, that is, when the diagnostic threshold falls within the range of ambiguous beliefs. When beliefs are ambiguous but q falls outside $\left[p_{i}^{L},p_{i}^{U}\right]$ such that $q<p_{i}^{L}$ or $p_{i}^{U}<q$ (i.e., PPH is likely or unlikely despite uncertainty), the decision about treatment is not affected by uncertainty. In other words, new information is valuable only when resolving uncertainty is necessary for decision-making.

We use d∈{0,1} to denote a patient's diagnosis and treatment status, where 0 indicates that the patient is not diagnosed with and treated for PPH, and 1 indicates that she is. Patient outcomes are given by:

$$\left\{ \begin{array}{c} 1,if\;\theta =0,d=0\\ 1-h,if\;\theta =0,d=1\\ 1-H,if\;\theta =1,d=0\\ 1-\delta H,if\;\theta =1,d=1 \end{array}\right.$$

Where 0≤1−H<1−δH<1−h<1, implying that treatment results in better outcomes for PPH patients, but PPH patients fare worse than non-PPH patients regardless of treatment. δ captures the (negative) effect of delays in treatment on patient outcomes; it increases in the time lapse between PPH onset and treatment and falls within the range of $\left(\frac{h}{H},1\right)$.

The expected outcome for any patient with Pr(θ=1)=p is:

$$V_{d=1}=p\left(1-\delta H\right)+\left(1-p\right)\left(1-h\right)$$

$$V_{d=0}=p\left(1-H\right)+\left(1-p\right)$$

If the provider chooses to actively monitor the patient, she receives l≥1 signals $\left\{x_{1},\ldots x_{l}\right\}$ conditional on the patient's underlying state θ and updates her beliefs. l denotes the number of data points the provider expects to collect before uncertainty is resolved (in other words, l is the expected number of times monitoring is performed). If the provider chooses wait-and-see, she receives a signal $x^{\prime }$ in some later period.

For simplicity, we assume that regardless of the action chosen by the provider, her beliefs eventually converge to the same $p\in \left\{{\underline{p}}_{i},{\bar{p}}_{i}\right\}$, where $p_{i}^{L}\leq {\underline{p}}_{i}<q\leq {\bar{p}}_{i}\leq p_{i}^{U}$. This implies that PPH will eventually be uncovered and will not resolve on its own, which is consistent with the progression of untreated PPH towards severe hemorrhage. If the provider's beliefs converge to some ${\underline{p}}_{i}$, she rules out PPH; if they converge to some ${\bar{p}}_{i}$, she diagnoses and treats the patient for PPH.

Let μ be the provider's perceived PPH prevalence (i.e., the number of patients out of 100 for whom the provider's beliefs converge to some $\bar{p}>q$), T the cost of treatment, and C the cost of conducting active monitoring. Denote δ under active monitoring with $\delta^{m}$ and δ under wait-and-see with $\delta^{w}$. The expected net payoff of active monitoring $V^{m}$ and that of waiting $V^{w}$ for patient i are:

$$V_{i}^{m}=\mu \times \left[{\bar{p}}_{i}\left(1-\delta^{m}H\right)+\left(1-{\bar{p}}_{i}\right)\left(1-h\right)-T\right]+\left(1-\mu \right)\times \left[{\underline{p}}_{i}\left(1-H\right)+\left(1-{\underline{p}}_{i}\right)\right]-C$$

$$V_{i}^{w}=\mu \times \left[{\bar{p}}_{i}\left(1-\delta^{w}H\right)+\left(1-{\bar{p}}_{i}\right)\left(1-h\right)-T\right]+\left(1-\mu \right)\times \left[{\underline{p}}_{i}\left(1-H\right)+\left(1-{\underline{p}}_{i}\right)\right]$$

The provider will actively monitor patient i if $V_{i}^{m}>V_{i}^{w}$, that is: $\mu \times {\bar{p}}_{i}\left(\delta^{w}-\delta^{m}\right)H>C$.

### 5.2 Model implications

#### 5.2.1 Implication 1: the provider will not actively monitor if additional information is perceived to be of little value

Information may not be valued by the provider under two scenarios. First, information is not valuable if the provider feels certain about her assessment of the patient's PPH risk, i.e., her belief consists of a single value $p_{i}$. Second, when uncertainty is present, information is not valuable if the perceived PPH risk is already low or high such that new information will not alter the provider's diagnosis, i.e., the diagnostic threshold q falls outside the bounds of ambiguous beliefs $\left[p_{i}^{L},p_{i}^{U}\right]$. In both situations, the provider could readily confirm or rule out PPH by comparing $p_{i}$ (or '$p_{i}$s in the case of ambiguous beliefs) against q without considering additional information. This establishes the first potential mechanism for under-monitoring. It could happen if e.g., the provider is overly precise in her risk assessment or PPH risk is grossly underestimated – in the model, the latter is represented by sets of ambiguous beliefs with the upper bound $p_{i}^{U}$ falling below q (we dismiss the possibility that PPH risk is grossly overestimated, as it would imply that many patients are treated for PPH, which is not what we have observed).

The model also generates additional implications regarding why under-monitoring could happen even when additional information is needed for a diagnosis. This concerns situations where the cost of monitoring outweighs its benefit, i.e., $\mu \times {\bar{p}}_{i}\left(\delta^{w}-\delta^{m}\right)H<C\text{.}$ In this case, the provider would prefer wait-and-see to active monitoring.

#### 5.2.2 Implication 2: if the perceived PPH prevalence μ is low, the provider is less likely to monitor

If μ is sufficiently low, the provider may monitor no patients at all. In this model, we assume that the diagnostic threshold q is given, but q need not reflect the “true” prevalence. For example, some women do not stay in the study facilities for the recommended 24 h after delivery and might develop PPH at home, which providers cannot observe. Since many PPH cases are identified shortly after delivery, as suggested in our data, it is also possible that providers (mistakenly or not) believe that PPH is very rare among patients who show no signs of hemorrhage immediately after delivery.

#### 5.2.3 Implication 3: if the perceived effect of delay in treatment $\left(\delta^{w}-\delta^{m}\right)H$ is small, the provider is less likely to monitor

This could be the case if e.g., the provider is indeed skilled at managing severe PPH or the provider is overconfident in her ability to manage PPH identified at a later stage. Since we make no assumptions about the kinds of adverse patient outcomes that matter most to the provider, it is also possible that the provider is only concerned about patient mortality, overlooking many severe morbidities (or “near-misses”) associated with PPH that could have been prevented if PPH was identified and managed early. When the provider only cares about preventing mortality, the perceived effect of treatment delays would be smaller, as maternal mortality is much rarer than near-misses (Owolabi et al., 2020).

#### 5.2.4 Implication 4: if the cost of active monitoring C is high, the provider is less likely to monitor

C captures the time and mental effort required to process information gathered through monitoring. In situations where other high-value activities such as attending to sick newborns compete for time and attention, C becomes more salient. While “wait-and-see” allows providers to receive highly informative signals, active monitoring may generate ambiguous signals perceived to be uninformative or difficult to interpret. Ambiguous signals result in more costly monitoring, as the number of data points l necessary to resolve uncertainty (uninformative signals) or the amount of time and effort required per monitoring (difficult-to-interpret signals) increases. If the provider believes the probability of receiving ambiguous signals to be high, she may not monitor at all. Alternatively, she may only perform checks that generate less ambiguous data, such as blood pressure monitoring, resulting in inconsistent adherence to guideline-recommended actions conditional on monitoring as reflected in our data.

In sum, our model implies that postpartum monitoring may be limited if providers do not perceive a need for additional information or if, upon perceiving information needs, providers prefer “wait-and-see” to active monitoring. This also implies that a lack of monitoring observed empirically may represent a situation where the possibility of PPH has been ruled out, but also one where the provider has yet to make a diagnosis but is instead waiting for information to emerge.

## 6 Discussion

The inconsistent progress towards reducing maternal mortality in Kenya and many other LMICs signals important gaps in the prevention, early detection, and management of preventable causes of maternal deaths (Keats et al., 2017; WHO, 2015). In this study, we showed that maternity care providers in three large teaching hospitals in Kenya performed very little monitoring of potential signs of PPH – an obstetric emergency responsible for one-fifth of maternal mortality across LMICs (Say et al., 2014) – despite a generally high level of knowledge about appropriate clinical practice. Because PPH often occurs in women without identifiable risk factors, the lack of routine monitoring could cause significant delays in detection of and response to PPH.

Our survey vignettes illustrated how the decision to actively monitor was linked to providers' subjective risk and perceived uncertainty. The results echoed some of the findings from formative interviews conducted by the study team. For example, during these interviews, one provider stated that “*I can say that most of the time the delays [in PPH management] will only occur mostly when you had already thought that the mother is stable, like hours later*.” Another provider mentioned “*some [PPH cases] go unidentified until a mother collapse[d] and that's when you realize, ‘What was the problem?’, then when you go you find she was bleeding a lot*.” Through these interviews, we also observed a tendency for providers to rely on patients and their companions to notice abnormal blood loss and call for help, which is in line with a “wait-and-see” approach. One provider also mentioned that inexperienced providers tend to wait for obvious signs of PPH before they suspect PPH.

Motivated by findings from the direct observations and vignettes, we introduced a simple model of provider decision-making under uncertainty to help explain limited postpartum monitoring. According to the model, a provider may not actively monitor a patient if she can confirm or rule out the possibility of PPH without additional information, or if she chooses to “wait and see” before drawing a conclusion about the patient's PPH state. In the first case, the provider does not perceive a need for new information. In the second case, the provider does perceive a need for information but decides to wait for information to emerge given her perception of PPH incidence, self-assessed ability to manage severe PPH, and perceived effort and time cost of monitoring.

Our study is concerned with why providers do not actively seek easily accessible information to make informed diagnostic decisions. It differs from studies of decision bias in that we do not assume the presence of specific biases in the provider's judgement, e.g., whether a lack of uncertainty is consistent with well-calibrated judgement or due to overconfidence. Classic theories of cognitive biases, such as representativeness heuristic and satisficing, may offer alternative explanations for insufficient monitoring. However, our model could be extended to accommodate certain biases such as overestimation of own performance.

This study has several limitations. First, we did not model the provider's belief updating process. Our model assumes that the provider stops monitoring when diagnostic uncertainty is resolved. The low monitoring frequency observed in the study facilities suggests that providers often rule out PPH after having observed just a few data points. There are many possible explanations for why providers stop monitoring so readily that we cannot evaluate empirically. A better understanding of why they stop monitoring is an important area for future research. Second, while the core of our model is that providers make an active and considered decision about whether to monitor a patient, it may be that provider decision-making in this high-stress environment is more of an automatic process (Wood and Rünger, 2016). Third, providers' responses to the vignettes may not fully reflect how they think and behave in more realistic settings, especially given how we defined and measured uncertainty and monitoring. Their intention to monitor may also be higher than usual due to the vignettes representing a higher risk pool or the Hawthorne effect, but such bias is less concerning given our focus on the relative level of monitoring. Fourth, we explored the decision-making of a unitary provider on a unitary patient, which abstracts away from team practice and the mix of patient profiles being cared for simultaneously. A related factor is the physical environment of the maternity department, which can cause gaps in communications between providers assigned to different roles or wards in the maternity department. Considerations of team-based care would be useful for studying how moral hazard in teams and ineffective handoffs might contribute to limited monitoring. Finally, our analysis examined three regional referral facilities with high patient volumes and may not generalize to other types of facilities.

## 7 Conclusion

Institutional delivery has been increasing across LMICs (Doctor et al., 2019), but poor quality of care hinders further reductions in maternal mortality, and existing policy instruments focusing on training, service readiness, and incentives appear insufficient. New approaches to improving postpartum care should consider whether providers actively seek diagnostic information or whether they simply choose to wait for information to emerge. Are providers overly precise in their risk judgement such that they do not see the value of more information? Are they underestimating the risk of PPH for most patients, including those with established risk factors? Are they underestimating the prevalence of severe PPH? Are they overestimating their ability to manage severe PPH when it does emerge? Is monitoring considered effortful yet uninformative? Our work shows that a better understanding of these factors inherent to the provider's decision-making process and how they result in deviations from the “optimal” clinical decision-making could potentially lead to more impactful interventions to improve the quality of care in the critical postpartum period.

## CRediT authorship contribution statement

**Dan Han:** Conceptualization, Methodology, Formal Analysis, Data Curation, Writing - Original Draft Preparation, Writing - Review & Editing. **Emma Clarke-Deelder:** Conceptualization, Methodology, Investigation, Data Curation, Project Administration, Funding Acquisition, Writing - Review & Editing. **Nora Miller:** Methodology, Investigation, Data Curation, Project Administration, Writing - Review & Editing. **Kennedy Opondo:** Methodology, Investigation, Data Curation, Project Administration, Writing - Review & Editing. **Thomas Burke:** Funding Acquisition, Writing - Review & Editing, Supervision. **Monica Oguttu:** Funding Acquisition, Writing - Review & Editing, Supervision. **Margaret McConnell:** Conceptualization, Methodology, Funding Acquisition, Writing - Review & Editing, Supervision. **Jessica Cohen:** Conceptualization, Methodology, Funding Acquisition, Writing - Review & Editing, Supervision.

## Data Availability

Data will be made available on request.
