# Supplementary material for: Health care provider decision-making and the quality of maternity care: An analysis of postpartum care in Kenyan hospitals
Source: Soc Sci Med. 2023 Aug;331:116071. doi: 10.1016/j.socscimed.2023.116071 (PMC10410252; doi:10.1016/j.socscimed.2023.116071)
Supplement: Multimedia component 1 [file mmc1.docx]

**Appendix**

**Part I: Tables and Figures**

**Table A1. Time when postpartum hemorrhage (PPH) was suspected in delivery observations**

|  | n (%) |
| --- | --- |
| ≤ 15 minutes after delivery | 22 (40.0%) |
| 16-30 minutes after delivery | 9 (16.4%) |
| 31-60 minutes after delivery | 1 (1.8%) |
| 61-120 minutes after delivery | 9 (16.4%) |
| >120 minutes after delivery | 12 (21.8%) |
| Unclear | 2 (3.6%) |
| Total number of suspected PPH cases | 55 |

Notes: Sample includes all patients for whom the postpartum period (the first 24 hours after delivery or through discharge) was observed. A patient was suspected of developing PPH if a health care provider indicated concern with or took actions to manage potentially excessive bleeding, as observed by the enumerators.

**Table A2. Measures of postpartum hemorrhage (PPH) risk factors using delivery observation data**

| **Risk factor** | **Measure** |
| --- | --- |
| Multiple gestation | Number of babies delivered ≥ 2 |
| High parity | Parity ≥ 5 |
| History of PPH | Patient self-report of PPH in the previous pregnancy |
| History of C-section | Patient self-report of C-section in the previous pregnancy |
| Bleeding during current pregnancy | Antenatal care (ANC) book or patient self-report bleeding in current pregnancy |
| Anemia | Hemoglobin < 10 g/dl; based on ANC book or hemoglobin test performed during initial exam at admission |
| Oxytocin exposure | Received at least 10 IU of oxytocin for labor augmentation and induction combined  Note: exposure to greater amount of oxytocin during labor and delivery is associated with a higher risk of uterine atony – the most common cause of PPH – due to prolonged oxytocin desensitization; we used 10 IU as the cutoff following the literature (Grotegut et al. 2011). |
| Suspected infection | Received antibiotics during labor |
| Prolonged labor | Any of the following (Bernitz et al. 2019; Neal et al. 2010; Nystedt and Hildingsson 2014):   1. Duration of second stage of labor (from dilation of 10 cm to delivery) > 3 hours if parity = 0 or > 2 hours if parity > 0 2. Dilation rate between dilation at admission and 10 cm < 0.5 cm dilated per hour if parity = 0 or < 1 cm per hour if parity > 0 3. Time from dilation of X cm at admission to delivery > 95 percentile of the distribution among all patients dilated at X cm at admission |
| Large baby (macrosomia) | Birthweight ≥ 4 kg |
| Suspected retained placenta | Time from delivery of fetus to placenta delivery ≥ 30 minutes |

Notes: The above risk factors are selected based on the literature, inputs from clinicians on the research team, the Kenyan guidelines on obstetric and newborn care, and available data. Higher degree laceration, a known PPH risk factor, is not included because there is not enough information on the degree of laceration at the time laceration was identified. If the data is missing because the health care provider did not ask the patient about it or perform the relevant health check, the risk factor is coded zero instead of missing, as we aim to capture “recorded” risk factors. If the data is missing because the enumerator did not observe the data, the risk factor is coded as missing.

**Table A3. Incidence of suspected postpartum hemorrghate (PPH) by documented risk factors**

| Number of PPH risk factors | All patients with complete observation | |  | Removed PPH cases detected within 15 minutes of delivery | |
| --- | --- | --- | --- | --- | --- |
|  | PPH incidence | Adj. difference relative to 0 risk factor |  | PPH incidence | Adj. difference relative to 0 risk factor |
| 0 | 0.081 | -- |  | 0.049 | -- |
|  |  |  |  |  |  |
|  |  |  |  |  |  |
| 1 | 0.079 | -0.013 |  | 0.059 | 0.004 |
|  |  | (0.025) |  |  | (0.022) |
|  |  |  |  |  |  |
| 2+ | 0.200 | 0.103* |  | 0.120 | 0.062 |
|  |  | (0.055) |  |  | (0.047) |
| Overall | 0.092 |  |  | 0.059 |  |

Notes: The difference in PPH incidence relative to patients with zero documented risk factor is adjusted for facility indicators and an indicator for referral cases. Sample includes patients with complete observation from admission through discharge. Suspected PPH is defined based on a provider’s indication of concern with or actions taken to manage potentially excessive bleeding, as observed by the enumerators. Robust standard errors in parentheses. * p < 0.1, ** p < 0.05, *** p < 0.01.

**Table A4. Fixed effects model estimates of average marginal effects of subjective PPH risk and indicated uncertainty on monitoring**

|  | Main model | Robustness 1: Responses assigned to brackets based on midpoint of risk interval | Robustness 2: Alternative definition of monitoring |
| --- | --- | --- | --- |
| Risk Bracket [2,3] vs. [0,1] | 0.075** | 0.111** | 0.127*** |
|  | (0.036) | (0.055) | (0.039) |
|  |  |  |  |
| Risk Bracket [4,5] vs. [0,1] | 0.315*** | 0.335*** | 0.269*** |
|  | (0.039) | (0.015) | (0.071) |
|  |  |  |  |
| Risk Bracket [6,7] vs. [0,1] | 0.306*** | 0.344*** | 0.244** |
|  | (0.039) | (0.048) | (0.103) |
|  |  |  |  |
| Risk Bracket [8,10] vs. [0,1] | 0.453*** | 0.522*** | 0.360*** |
|  | (0.044) | (0.049) | (0.061) |
|  |  |  |  |
| Uncertainty 1 vs. 0 | 0.152** | 0.140*** | 0.144** |
|  | (0.060) | (0.053) | (0.066) |
|  |  |  |  |
| Risk Bracket [0,1]: Uncertainty 1 vs. 0 | 0.016 | -0.062 | -0.049 |
|  | (0.080) | (0.060) | (0.040) |
|  |  |  |  |
| Risk Bracket [2,3]: Uncertainty 1 vs. 0 | 0.270** | 0.294*** | 0.265*** |
|  | (0.124) | (0.105) | (0.058) |
|  |  |  |  |
| Risk Bracket [4,5]: Uncertainty 1 vs. 0 | 0.242*** | 0.196*** | 0.266** |
|  | (0.042) | (0.071) | (0.112) |
|  |  |  |  |
| Risk Bracket [6,7]: Uncertainty 1 vs. 0 | 0.247*** | 0.230*** | 0.277*** |
|  | (0.090) | (0.070) | (0.095) |
|  |  |  |  |
| Risk Bracket [8,10]: Uncertainty 1 vs. 0 | 0.014 | 0.085 | 0.006 |
|  | (0.206) | (0.134) | (0.056) |
| N | 576 | 576 | 576 |

Notes: Based on provider response to survey vignettes. This table reports the average marginal effects of subjective risk, indicated uncertainty in risk assessment, and the interaction of these two variables on the probability of a provider choosing to actively monitor the patient presented in a vignette. The coefficients reported represent the average difference in predicted probability of monitoring between a group and the reference group (i.e., risk bracket = [0,1] or uncertainty = 0). For example, the estimates for “Risk Bracket [4,5]: Uncertainty 1 vs. 0” represent the difference in the probability of active monitoring when uncertainty was indicated versus when it was not indicated for cases with a subjective PPH risk of 4-5. Results are based on linear probability models adjusted for provider fixed effects and vignette fixed effects. The dependent variable is a binary variable indicating whether a provider chose to monitor the patient described in a vignette. A provider indicated uncertainty if they specified a risk interval with lower and upper bounds instead of a point risk. The main model groups the data into risk brackets based on the lower bound of the risk interval; the robustness analysis uses the midpoint of the interval to determine the risk bracket. For the second robustness analysis, the outcome variable equals 1 if a patient was assigned to a bed in the post-delivery unit or the bed closest to the nurse station in the postpartum unit. Standard errors (in parentheses) are clustered at the facility level. * p < 0.1, ** p < 0.05, *** p < 0.01.

**Figure A1. Correlations between provider characteristics and the propensity to indicate uncertainty**

Notes: Based on provider response to survey vignettes. This figure reports the estimated coefficients on provider characteristics based on a linear probability model that regresses an indicator variable for whether a provider ever indicated uncertainty for any vignette on provider characteristics and facility indicators. 95% Cis are based on standard errors clustered at the facility level. ^+^Data on highest education and years of experience were not collected from medical students. * p < 0.1, ** p < 0.05, *** p < 0.01.

**Part II: Survey vignettes**

**Visual aids: patient card, facility map, and PPH risk scale**

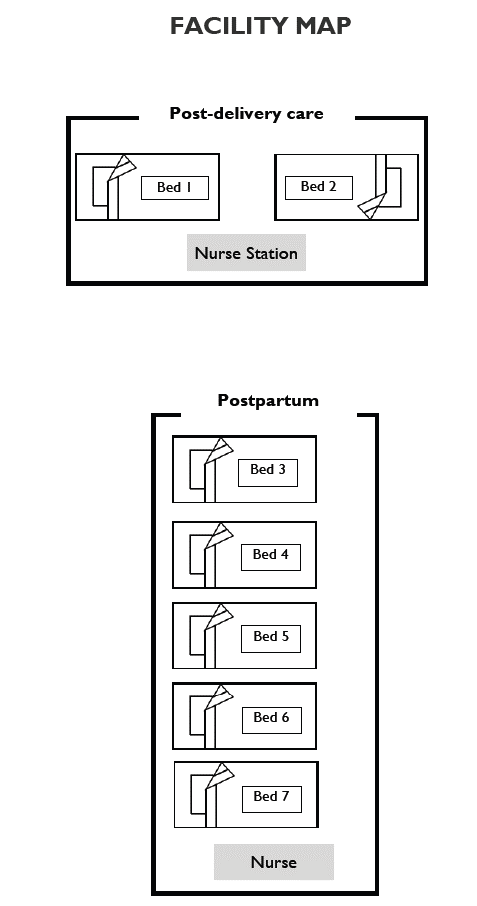

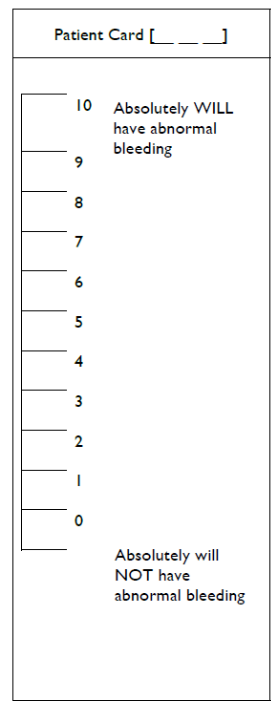


**Interview guide**

**Appendix Reference**

Bernitz, Stine et al. 2019. “The Frequency of Intrapartum Caesarean Section Use with the WHO Partograph versus Zhang’s Guideline in the Labour Progression Study (LaPS): A Multicentre, Cluster-Randomised Controlled Trial.” *The Lancet* 393(10169): 340–48.

Grotegut, Chad A. et al. 2011. “Oxytocin Exposure during Labor among Women with Postpartum Hemorrhage Secondary to Uterine Atony.” *American Journal of Obstetrics and Gynecology* 204(1): 56.e1-6.

Neal, Jeremy L. et al. 2010. “What Is the Slowest-Yet-Normal Cervical Dilation Rate Among Nulliparous Women With Spontaneous Labor Onset?” *Journal of Obstetric, Gynecologic & Neonatal Nursing* 39(4): 361–69.

Nystedt, Astrid, and Ingegerd Hildingsson. 2014. “Diverse Definitions of Prolonged Labour and Its Consequences with Sometimes Subsequent Inappropriate Treatment.” *BMC Pregnancy and Childbirth* 14(1): 233.
